# False Positive Positron Emission Tomography / Computed Tomography Scans in Treated Head and Neck Cancers

**DOI:** 10.7759/cureus.1146

**Published:** 2017-04-10

**Authors:** Michael K Cheung, Shawn Y Ong, Uma Goyal, Betsy C Wertheim, Charles C Hsu, Sun K Yi

**Affiliations:** 1 Radiation Oncology, University of Arizona, Tucson, AZ; 2 Arizona Cancer Center, University of Arizona, Tucson, AZ

**Keywords:** pet/ct, false positives, head and neck cancer, post-treatment, radiation therapy

## Abstract

**Objective:**

Positron emission tomography/computed tomography (PET/CT) imaging for head and neck cancers (HNC) is commonly utilized for post-treatment assessment. Though PET/CT in this setting has been reported to have high negative predictive values (> 90%), positive predictive values have been reported at approximately 50%, leading to high rates of false positivity (FP) and troubling management decisions for both patient and practitioner. The objective of this study was to identify patient, disease, treatment and imaging factors that might be associated with a higher likelihood of FP on initial post-treatment PET/CT imaging for patients treated for HNC.

**Materials and methods:**

A retrospective chart review was performed on 84 patients treated for HNC who received radiation therapy (RT) as part of their overall management from October 2005 to August 2013. Of the patients screened, 19 were found to have mucosally based squamous cell carcinoma (SCC) with positive initial post-treatment PET/CT studies (23%). Fisher’s exact test was used to analyze the association between categorical variables and FP, including patient's gender, disease laterality, primary tumor site and stage, nodal and overall stage, high dose RT fraction size, number of RT fractions completed, total RT dose, biologically effective dose and timing of PET/CT acquisition. Wilcoxon rank-sum test was used to analyze the association between continuous variables and FP, including patient age, total elapsed days of RT, an amount of infused fluorodeoxyglucose ^18^F-FDG, pre-PET/CT serum glucose levels, and maximum standardized uptake value SUV_max_. Statistically significant findings were those that were deemed p <0.05.

**Results:**

Among patients with positive initial post-treatment PET/CT scans for treated HNC, there was a lower proportion of higher primary disease stage associated with FP versus true positivity (T-stage 3-4: 20 vs 78%, respectively, *p=0.023*). We also discovered that 50% of patients that underwent confirmation for FP findings suffered serious complications as a direct consequence of invasive exploratory procedures.

**Conclusions:**

Although PET/CT is known for its exceptional negative predictive value (> 90%) in the post-treatment setting for HNC, high rates of FP remains a clinical challenge. Our study suggests that tumor stage (T-stage) may impact FP rates in positive initial post-treatment PET/CT scans. We recommend careful multidisciplinary discussion regarding positive PET/CT studies in the post-treatment setting for HNC, particularly if invasive intervention is considered.

## Introduction

It is estimated that over 48,000 new cases of head and neck cancer (HNC) were diagnosed in the United States (US) in 2016 [1]. Patients with HNC commonly present with locally advanced disease, requiring a multidisciplinary approach for optimal outcomes, with radiation therapy (RT) delivered either in the post-operative [2] or definitive setting [3]. Despite improvements in therapy, loco-regional failures (LRF) remain a substantial problem and often lead to cancer-related death [4]. Although options for the persistent or recurrent disease are limited and associated with low success rates [5], early detection of LRF has been shown to improve the ability for salvage [6].

Imaging is often acquired during the post-treatment assessment period and findings suggestive of LRF can be very clear and corroborated with gross disease found on physical and/or endoscopic examination. Biopsy should be obtained in these settings for histologic confirmation prior to further management. At other times, positive findings on post-treatment imaging may be less clear for LRF, particularly when highly sensitive imaging is used and non-descript post-treatment changes, including inflammation and fibrosis, may confound the diagnosis. Deciding upon invasive confirmatory procedures for positive findings, in these more ambiguous scenarios should be done with caution, as invasive procedures within irradiated fields are known to be associated with morbidities [7-8].

Positron emission tomography/computed tomography (PET/CT), which offers improved accuracy of disease detection over conventional techniques of imaging [9], is becoming increasingly utilized for HNC post-treatment surveillance [10-11]. Although negative predictive values (NPV) greater than 90% are consistently reported when PET/CT is utilized for post-treatment Head and Neck Cancers (HNC) surveillance, positive predictive values (PPV) have been as low as 50%, suggesting that false positives (FP) remain a challenge to physicians when management decisions are needed [12-13]. Therefore, we have performed a retrospective study to determine factors that are associated with false positivity in post-treatment PET/CT imaging.

## Materials and methods

### Patients

After approval by the University of Arizona institutional review board (1300000696), a retrospective chart review was performed on HNC patients treated from October 2005 to August 2013 who received RT as part of their overall management. Patients treated by multidisciplinary effort including surgical resection and/or systemic therapy were included. We included only patients diagnosed with mucosal squamous cell carcinoma (SCC) and excluded any patients presenting with distant metastatic disease. Patients were not required to have an initial diagnostic pre-treatment PET/CT but were required to have had PET/CT imaging as part of their post-treatment assessment.

From a total of 84 patients with HNC screened, 73 were discovered to have SCC histology, and 71 patients with the mucosal-based disease. From those remaining patients, 19 were found with positive findings in the irradiated head and neck region on initial post-treatment PET/CT scans. 

### Treatment planning

Simulation procedure was performed with a head, neck, and shoulder thermoplastic mask Type-S Head, Neck & Shoulder Perforated Mask (CIVCO Medical Solutions, Coralville, IA, United States) with the patient’s head cradled within a resting device (Timo Headrest, CIVCO Medical Solutions, Coralville, IA, United States). CT simulation imaging was acquired with a Philips Brilliance Big Bore scanner (Andover, Massachusetts, United States) in 3 mm slices from skull vertex to mid-thorax without and/or with the administration of Isovue contrast (Bracco Imaging, Monroe Township, New Jersey, United states) based on appropriate kidney function. All patients were treated with external beam radiation therapy (EBRT) with 3D conformal or intensity-modulated radiotherapy (IMRT) technique. Patients were treated with either sequential or simultaneous integrated boost technique for differential dosing to high, intermediate, and low-risk regions depending on findings at the time of diagnosis if treated definitively or upon pathologic evaluation in the post-operative setting. 

For patients treated in the definitive setting, gross tumor volume (GTV) was delineated with the assistance of diagnostic imaging. A 5 mm uniform margin was then applied to the GTV utilizing the treatment planning software auto-expansion tool to create a clinical target volume (CTV) to account for the microscopic spread. The CTV was edited from normal anatomic boundaries and an additional margin of 3-5 mm was added for the planned target volume (PTV) to account for daily set-up uncertainty and physiologic motion. Intermediate- and low-risk neck PTVs were also created based on the primary site of origin and its potential pattern of lymphatic failure based on previous publications [14-15]. Normal tissues were contoured including optic nerves and chiasm, brainstem with 3 mm expansion, bilateral cochleas, spinal cord with 5 mm expansion, bilateral parotid glands, lips, oral cavity, mandible, superior constrictor muscles, larynx, esophagus, and bilateral brachial plexus. During the planning process, a D95 target coverage was applied for each PTV per international commission on radiation units and measurements (ICRU 62) recommendations and normal tissues were constrained according to well-accepted guidelines [16]. A similar process for treatment planning except for GTV was undertaken in the post-operative setting, though areas of resected gross disease constituted the highest risk PTV, which was delineated after careful review of pre-surgical imaging, operative and pathologic reports.

### Radiation therapy

Seventeen patients were treated with definitive intent RT and two patients were treated post-operatively. All patients were treated with once daily high-dose RT fraction sizes between 1.8 Gy to 2.12 Gy, except one patient who was treated with definitive intent RT utilizing 1.25 Gy twice daily fractionation. The median duration of RT for patients treated in the definitive and post-operative setting was 51 elapsed days (range: 39–67 days) and 54.5 elapsed days (range: 54–55 days) respectively. The median number of treatment fractions delivered for patients in the definitive and post-operative setting was 35 (range: 33–61) and 35 (range: 33–37), respectively. Patients who were treated with definitive intent received a median dose of 70 Gy (range: 66–76.25 Gy) to the planning target volume (PTV) encompassing gross disease, while post-operative patients received a median dose of 66.3 Gy (range: 66–66.6 Gy) to the PTV region encompassing previously resected gross disease. RT doses were then converted into BED_3_ and BED_10_ with the following equation: BEDx = nd (1 + d/ α/β); where x = three or 10, n = total number of fractions delivered, d = fractional RT dose in Gy, and α/β = three or 10, respectively. The median BED_3_ for patients who received RT in the definitive setting was 117 Gy (range: 108–127 Gy), and for post-operative patients, the median BED_3_ was 108 Gy (range: 107–110 Gy). The median BED_10_ for patients who received RT in the definitive setting was 84 Gy (range: 79–91 Gy), and for post-operative patients, the median BED_10_ was 79 Gy.

### Post-treatment PET/CT assessment

Prior to November 2010, 13 patients were referred to outside institutions for post-treatment PET/CT imaging. The remaining six patients underwent PET/CT scanning at our institution utilizing a standardized protocol. PET/CT scans were obtained following four-six hours of fasting after serum glucose levels were found to be < 175 mg/dL. Sixty +/- 10 minutes following IV administration of ^18^F-FDG at 0.1 mCi/kg, sequential PET and CT imaging was performed with an integrated General Electric (GE) discovery 690 PET/CT scanner (Little Chalfont, United Kingdom) with a time of flight algorithm from skull vertex to mid-thigh. Helical CT imaging was taken with 120 kVp, 1.5 mm collimation and 0.94 pitch parameters. Images were then interpreted by a board-certified nuclear medicine physician using software with fusion capability (Mirada Medical, Denver, Colorado, United States).

Positive findings in post-treatment PET/CT scans were defined as those with standardized uptake value (SUV) max >2.0 within irradiated volumes of the head and neck with or without CT correlates. Positive findings were discussed within a multidisciplinary setting and recommendations for further workup included repeat serial imaging and clinical examination or histologic diagnosis with biopsy. Findings that were positive on post-treatment PET/CT that were eventually discovered negative for disease recurrence were considered FP.

### Statistical methods

Categorical variables including patient gender, disease laterality, primary tumor site, primary tumor (T) stage, nodal (N) stage, overall stage, high dose RT fraction size, number of RT fractions completed, total RT dose, biologically effective dose (BED)_10_ > 84 vs. ≤ 84, BED_3_ > 110 vs. ≤ 110, timing of PET/CT acquisition (> 90 vs. ≤ 90 days), and positive findings within or outside original areas of primary or nodal disease prior to treatment and their associations with FP findings on post-treatment PET/CT were evaluated using Fisher’s exact tests. Continuous variables including patient age, total elapsed days of RT, amount of infused ^18^F-FDG (mCi), pre-PET/CT serum glucose (mg/dL) measurements and SUV_max_ were analyzed for associations with FP using Wilcoxon rank-sum tests. Statistically significant associations were reported with p-values < 0.05. No corrections were made for multiple comparisons. All statistical analyses were performed using Stata 14.1 (StataCorp, College Station, Texas, united States).

## Results

### Patient and disease characteristics

Fifteen males and four females with a median age of 65 years (range: 42-79 years) with HNC of mucosal SCC histology treated with RT as part of their overall management and with positive post-treatment PET/CT were analyzed. Table 1 provides a summary of patient and disease characteristics.

**Table 1:** Patient and disease characteristics

Characteristic	Value – *n* (%)
Age (yrs)	
Median	65
Range	42 – 79
Gender	
Male	15 (79)
Female	4 (21)
Smoking History (>10 pk-yr)	
Yes	10 (53)
No	9 (47)
Tumor Site	
Oral Cavity	2 (11)
Oropharynx	6 (32)
Larynx	8 (42)
Hypopharynx	2 (11)
Nasal Cavity/Sinuses	1 (5)
AJCC Overall Stage	
Stage I	1 (5)
Stage II	2 (11)
Stage III	4 (21)
Stage IVA	10 (53)
Stage IVB	2 (11)
Treatment	
Definitive	17 (89)
Post-Operative	2 (11)
Concurrent Chemotherapy	
Platinum based	12 (63)
None	7 (37)
Follow-up (mos)	
Median	13
Range	4 – 84

Seventy-nine percent of patients were male. Fifty-three percent had greater than 10 pack-years of smoking history. The most frequently treated primary site was larynx (42%), followed by oropharynx (32%), oral cavity (11%), hypopharynx (11%), and nasal cavity/sinuses (five percent). Fifty-three percent of patients were stage IVA, followed by stage III (21%), stage IVB (11%), stage II (11%) and stage I (five percent). Sixty-three percent patients were treated with definitive intent RT concurrent with platinum-based chemotherapy. Median follow-up was 13 months (range: four to 84 months) and all patients received post-treatment PET/CT imaging.

### Post-treatment PET/CT findings

**Figure 1:**
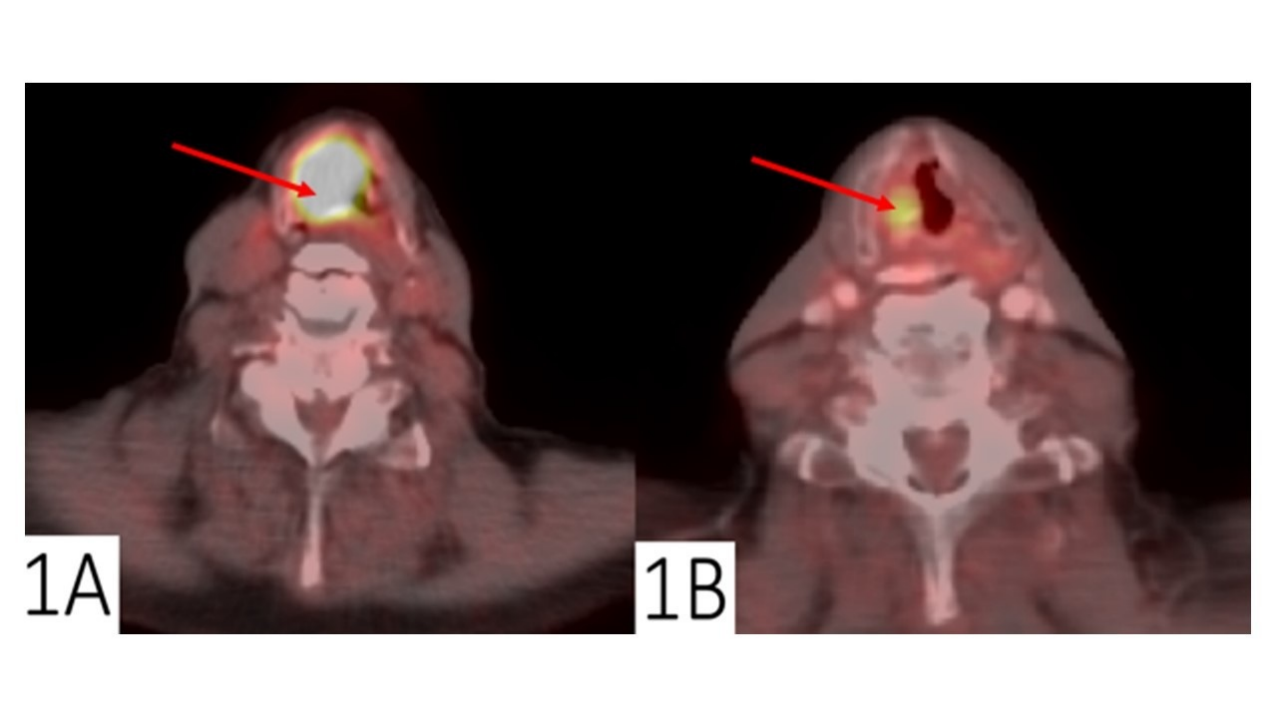
True positive post-treatment positron emission tomography/computed tomography (PET/CT) scan Pre-treatment and post-treatment positron emission tomography/computed tomography (PET/CT) for a laryngeal cancer. Representative matched axial PET/CT scan slices at approximately the level of the true glottis from a patient treated with definitive chemoradiation for initially staged T3N0M0, stage III, laryngeal cancer; (a) pre-treatment PET/CT scan with a CT mass measuring 2.7 x 2.3 cm at this level and SUVmax of 14 and; (b) post-treatment PET/CT scan with a residual CT mass measuring 1.4 x 1.1 cm and SUVmax of 5.9

All 19 patients received post-treatment PET/CT imaging. The median time interval between the completion of RT and the initial post-treatment PET/CT scan for all patients was 95 days (range: 28–711 days). Nine patients were confirmed to have LRF and therefore true positive (TP) findings on post-treatment PET/CT imaging. The median time interval between completion of RT and post-treatment PET/CT scan for patients with TP was 95 days (range: 65–711 days). Patients with TP were determined to have LRF by histologic confirmation (five patients), serial clinical examination (three patients), or repeat imaging (one patient). Two of the nine patients with TP findings had CT correlates within the areas of elevated SUV on PET, which corresponded to areas of known pre-treatment disease. One patient with a TP post-treatment PET/CT, initially presented with a T3N0M0, stage III, right laryngeal SCC (Figure 1), had an incomplete response to definitive chemoRT and underwent salvage laryngectomy, but died of his disease shortly thereafter.

**Figure 2:**
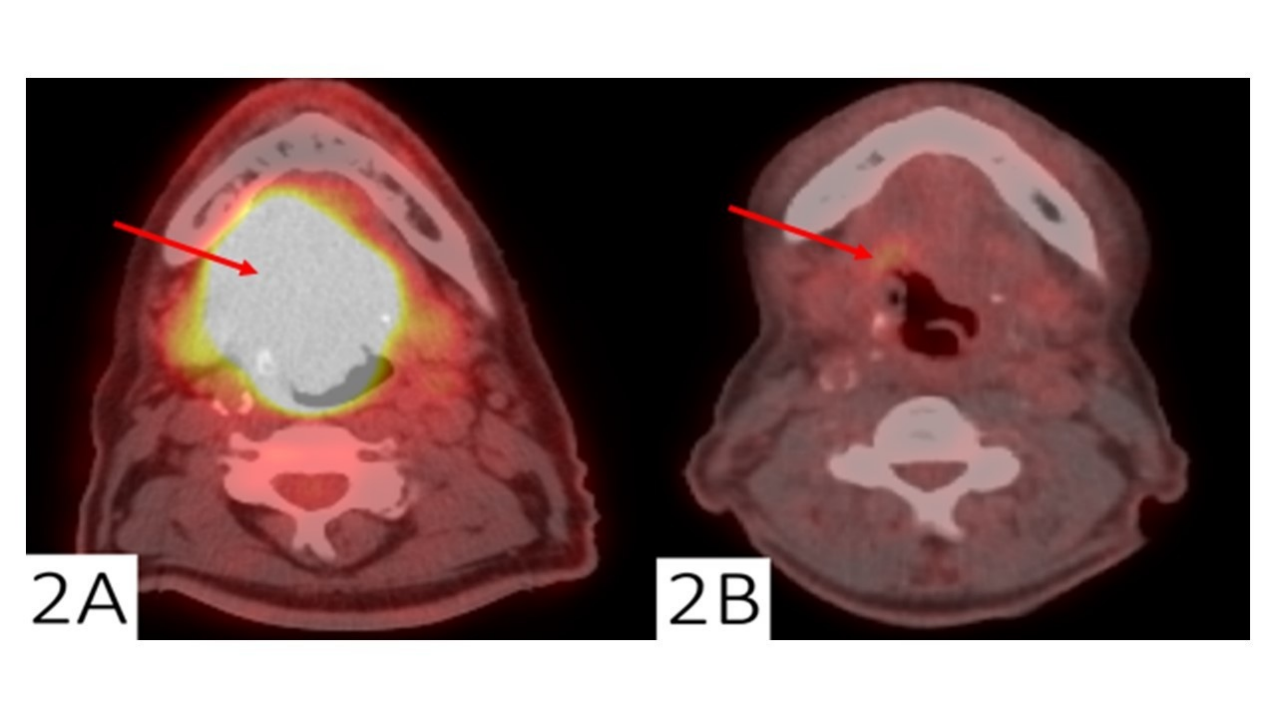
False positive post-treatment positron emission tomography/computed tomography (PET/CT) scan Pre-treatment and post-treatment positron emission tomography/computed tomography (PET/CT) for an oropharyngeal cancer. Representative matched axial PET/CT scan slices at approximately the level of the base of tongue from a patient treated with definitive chemoradiation for initially staged T4aN0M0, stage IVA, oropharyngeal cancer; (a) pre-treatment PET/CT scan with a CT mass measuring 5.7 x 5.0 cm at this level and SUVmax of 18.2 and; (b) post-treatment PET/CT scan with no obvious residual CT mass and SUVmax of 4.3

Ten patients were determined to have a FP finding on their initial post-treatment PET/CT study. The median time interval between completion of RT and post-treatment PET/CT scan was 89.5 days (range: 28–350 days). One patient was followed clinically without LRF, five were serially imaged with resolution of initially positive findings, and four underwent histologic confirmation with negative biopsies for LRF. None of the 10 patients with FP findings had CT correlates in the areas of elevated SUV. Two of the four patients with FP findings that underwent biopsy had a direct complication of the invasive diagnostic procedure, including one patient who suffered laryngeal edema after direct laryngoscopy requiring an emergent cricothyroidotomy followed by permanent tracheostomy. The other patient suffered post-biopsy hyoid osteomyelitis and despite a prolonged course of antibiotics, subsequently decompensated in speech and swallowing function (Figure 2).

The positive predictive value (PPV) of initial post-treatment PET/CT surveillance for our cohort was therefore 47%.

### Factors associated with post-treatment PET/CT false positive findings

Patient, disease, treatment, and imaging characteristics of patients who had TP versus FP findings on initial post-treatment PET/CT are described in Tables 2-3.

**Table 2:** Associations with false positive positron emission tomography/computed tomography (PET/CT) findings for categorical variables

Variable	True Positive * n* = 9 (%)	False Positive * n* = 10 (%)	*p*-value^1^
Gender			0.667
Female	2 (22)	2 (20)	
Male	7 (78)	8 (80)	
Laterality			0.370
Left	2 (22)	4 (40)	
Right	7 (78)	6 (60)	
Primary tumor site			0.111
Larynx	6 (67)	2 (20)	
Oropharynx	1 (11)	5 (50)	
Other	2 (22)	3 (30)	
T stage			0.023
1–2	2 (22)	8 (80)	
3–4	7 (78)	2 (20)	
N stage			0.370
0–1	6 (67)	4 (40)	
2–3	3 (33)	6 (60)	
Overall stage			0.650
I–III	4 (44)	3 (30)	
IV	5 (56)	7 (70)	
High dose fraction			0.582
≤ 2 Gy	8 (89)	7 (70)	
> 2 Gy	1 (11)	3 (30)	
Fractions completed			1.000
< 35	3 (33)	3 (30)	
≥ 35	6 (67)	7 (70)	
Total RT dose			0.650
< 70 Gy	4 (44)	3 (30)	
≥ 70 Gy	5 (56)	7 (70)	
BED_10_			0.141
≤ 84	8 (89)	5 (50)	
> 84	1 (11)	5 (50)	
BED_3_			0.303
≤ 110	3 (33)	1 (10)	
> 110	6 (67)	9 (90)	
Timing of PET/CT post-RT			0.170
≤ 90 days	2 (22)	6 (60)	
> 90 days	7 (78)	4 (40)	
Original areas of disease			0.582
No	1 (11)	3 (30)	
Yes	8 (89)	7 (70)	

**Table 3:** Associations with false positive positron emission tomography/computed tomography (PET/CT) findings for continuous variables: median interquartile range (IQR)

Variable	True Positive *n* = 9	False Positive *n* = 10	*p*-value^1^
Age (yrs)	65 (51–66)	63 (58–71)	0.306
Total elapsed days of RT	55 (53–60)	50 (49–51)	0.177
Pre-PET/CT serum glucose (mg/dl)	116 (106–127)	110.5 (93–121)	0.513
PET/CT ^18^F-FDG (mCi)	10.1 (8.5–10.5)	11.2 (8.8–12)	0.236
PET/CT SUV_max_	5.25 (4.3–6)	4.15 (2.7–7.3)	0.328

There was a lower proportion of higher T-stage primary disease in patients found with FP compared with TP post-treatment PET/CT scans (T-stage 3-4: 20 vs 78%, respectively, p=0.023). No other statistically significant results were found among the remaining variables.

## Discussion

PET/CT is an effective imaging modality for detecting disease in patients that have been previously treated for HNC [10-11]. Although PET/CT scanning is known to have an excellent negative predictive value (NPV) for post-treatment HNC assessment, low PPV in the 50% range leads to troubling FP findings, which are often difficult for practitioners to manage [12-13]. In our small cohort of 19 patients with positive findings on initial post-treatment PET/CT, we found a PPV of 47%, which is in keeping with that described in the literature.

When positive results on the initial post-treatment PET/CT are discovered with gross disease persistence or recurrence found on clinical examination, there is a general consensus that prompt histologic confirmation that should be obtained by tissue biopsy, as early salvage therapy has been shown to improve patient outcomes [6]. When positive findings on the initial post-treatment PET/CT are more equivocal or ambiguous; however, the decision to biopsy for disease confirmation becomes less clear as the inherent risks associated with invasive diagnostic intervention may potentially outweigh its benefits [17]. This is particularly true for tissue that is at increased risk for poor wound healing, necrosis or infection has given previous irradiation [7-8]. In our study, 50% of patients that underwent invasive confirmatory procedures for FP findings on initial post-treatment PET/CT suffered serious complications as a direct consequence of their tissue biopsy. 

The objective of our study was to identify patient, disease, treatment, and imaging factors that might be associated with a higher likelihood of false positivity on initial post-treatment PET/CT scan for HNC patients. To our knowledge, no other study to date has been published focusing on this particular phenomenon. We found that among patients with FP versus TP findings on post-treatment PET/CT, there was a lower proportion of higher primary disease stage (T-stage 3-4: 20 vs 78%, respectively, p=0.023). It is well known that advanced T-stage is not only a prognostic factor but also predictive of LRF in patients treated for HNC [18]. Patients with lower primary disease stage (T-stage 1-2) and positive post-treatment PET/CT scans may, therefore, be at higher risk for FP, because elevated SUV in the irradiated regions may be associated with persistent inflammatory changes within the surrounding normal tissue and tumor bed rather than malignancy. Inflammation induced by RT is well described [19-20], although factors affecting persistent ^18^F-FDG uptake and FP rates on post-treatment PET/CT scans are not well understood.

Some have suggested that the timing of PET/CT acquisition post-treatment may play a role in inaccurate findings and that a minimum of three months should pass before surveillance imaging is pursued [21-25]. Our study showed that timing of PET/CT acquisition when categorically evaluated as taken before or after 90 days post-treatment was not associated with FP findings (p=0.170). Other parameters we evaluated have previously been documented as having an RT dose relationship and increasing incidence with higher BED, including dermatitis and mucositis, which are thought to be immune/inflammatory modulated processes [26-27]. In our study, larger than conventional fraction RT dose (> 2 Gy), total RT dose (≥ 70 Gy), and higher BEDs did not show statistically significant associations with FP findings.

Variable techniques in PET/CT acquisition and interpretation are known to exist across institutions which may impact the accuracy of post-treatment PET/CT imaging for treated HNC patients. Specific machine parameters such as scanner resolution and partial volume effects, in addition to patient pre-PET/CT serum glucose measurements, amount of ^18^F-FDG activity injected, lean body mass or body surface area, timing from injection of ^18^F-FDG tracer to image acquisition, ^18^F-FDG tumor and tissue metabolism kinetics and region of interest demarcation, among other factors are thought to effect post-treatment PET/CT interpretation [28]. Our analysis showed that there were no significant differences in pre-PET/CT serum glucose, an amount of ^18^F-FDG activity injected, and SUV_max_ between patients found with FP versus TP on post-treatment PET/CT imaging.

A substantial proportion of the patients treated for HNC suffers heightened anxiety during the surveillance period after curative management [29-30]. It is not surprising that if a positive finding is discovered on post-treatment PET/CT imaging, both physician and patient may become distressed. Multidisciplinary discussion, however, should be held on whether invasive intervention should be pursued as the first approach for narrowing the diagnosis, as our data and others have shown 50% PPV in these circumstances. One might consider instead, other methods of imaging including diffusion weighted magnetic resonance imaging (MRI), dynamic contrast-enhanced MRI, or evaluation of metabolic kinetics of post-treatment PET/CT.

Our analysis is limited by its retrospective nature and the small number of positive post-treatment PET/CT events. Out of a total of 84 patients with HNC initially screened from our institution, only 19 were found to have mucosally-based SCC with positive post-treatment PET/CT studies (23%). Additionally, some of our post-treatment PET/CT studies were performed at outside facilities, due to lack of PET/CT capability at our institution prior to November 2010. As a result, unfortunately, PET/CT technique parameters other than pre-PET/CT serum glucose, an amount of ^18^F-FDG activity injected and SUV_max_ were not available for evaluation in patients scanned out the outside facilities.

## Conclusions

Although PET/CT is increasingly utilized for post-treatment HNC assessment with excellent reported NPVs in the literature (> 90%), PPVs associated with post-treatment PET/CT scanning for this population of patients is dismal at approximately 50%, which results in high rates of FP findings, as shown in this study. After curative management of HNC, a positive finding on initial post-treatment imaging is disconcerting for both patient and practitioners alike. Options for further management are wide ranging and could include invasive salvage or diagnostic interventions, which may lead to complications that were resultant to interventions performed unnecessarily. Fifty percent of patients in our study who were found with FP post-treatment PET/CT scans that underwent invasive procedures suffered significant morbidity. Therefore, we sought to find factors that might be associated with increased FP rates on post-treatment PET/CT for treated HNC, in order to provide guidance to practitioners found in this quandary. Based on our results we found an association with the lower T-stage disease with increasing FP on initial post-treatment PET/CT scans for treated HNC.

## References

[1] Siegel RL, Miller KD, Jemal A (2016). Cancer statistics, 2016. CA Cancer J Clin.

[2] Bernier J, Cooper JS, Pajak TF (2005). Defining risk levels in locally advanced head and neck cancers: a comparative analysis of concurrent postoperative radiation plus chemotherapy trials of the EORTC (# 22931) and RTOG (# 9501). Head Neck.

[3] Ang KK, Harris J, Wheeler R (2010). Human papillomavirus and survival of patients with oropharyngeal cancer. N Engl J Med.

[4] Pignon J-P, Le Maitre A, Maillard E (2009). Meta-analysis of chemotherapy in head and neck cancer (MACH-NC): an update on 93 randomised trials and 17,346 patients. Radiother Oncol.

[5] Mabanta SR, Mendenhall WM, Stringer SP (1999). Salvage treatment for neck recurrence after irradiation alone for head and neck squamous cell carcinoma with clinically positive neck nodes. Head Neck.

[6] Wong LY, Wei WI, Lam LK (2003). Salvage of recurrent head and neck squamous cell carcinoma after primary curative surgery. Head Neck.

[7] Yi SK, Kratz S, Gernon TJ (2017). Morbidity associated with false-positive findings in post-treatment positron emission tomography/computed tomography in chemoradiation treated head and neck cancer patients. Jacobs J Radiat Oncol.

[8] Tibbs MK (1997). Wound healing following radiation therapy: a review. Radiother Oncol.

[9] Adams S, Baum RP, Stuckensen T (1998). Prospective comparison of 18F-FDG PET with conventional imaging modalities (CT, MRI, US) in lymph node staging of head and neck cancer. Eur J Nucl Med.

[10] Kostakoglu L, Fardanesh R, Posner M (2013). Early detection of recurrent disease by FDG-PET/CT leads to management changes in patients with squamous cell cancer of the head and neck. Oncologist.

[11] Heron DE, Andrade RS, Beriwal S (2008). PET-CT in radiation oncology: the impact on diagnosis, treatment planning, and assessment of treatment response. Am J Clin Oncol.

[12] Patel K, Hadar N, Lee J (2013). Lack of evidence for PET or PET/CT surveillance of patients with treated lymphoma, colorectal cancer, and head and neck cancer: a systematic review. J Nucl Med.

[13] Gupta T, Master Z, Kannan S (2011). Diagnostic performance of post-treatment FDG PET or FDG PET/CT imaging in head and neck cancer: a systematic review and meta-analysis. Eur J Nucl Med Mol Imaging.

[14] Chao KC, Ozyigit G, Tran BN (2003). Patterns of failure in patients receiving definitive and postoperative IMRT for head-and-neck cancer. Int J Radiat Oncol Biol Phys.

[15] (2017). CT- based delineation of lymph node levels in the N0 neck: DAHANCA, EORTC, GORTEC, RTOG consensus guidelines. http://www.rtog.org/LinkClick.aspx?fileticket=TjrmNiHXly8%3d&tabid=229.n.d..

[16] Marks LB, Yorke ED, Jackson A (2012). Use of normal tissue complication probability models in the clinic. Int J Radiat Oncol Biol Phys.

[17] Batsakis JG, Sneige N, El-Naggar AL (1992). Fine-needle aspiration of salivary glands: its utility and tissue effects. Ann Otol Rhinol Laryngol.

[18] Leemans CR, Tiwari R, Nauta JJ (1994). Recurrence at the primary site in head and neck cancer and the significance of neck lymph node metastases as a prognostic factor. Cancer.

[19] Zhao W, Robbins ME (2009). Inflammation and chronic oxidative stress in radiation-induced late normal tissue injury: therapeutic implications. Curr Med Chem.

[20] Lorimore SA, Coates PJ, Scobie GE (2001). Inflammatory-type responses after exposure to ionizing radiation in vivo: a mechanism for radiation-induced bystander effects?. Oncogene.

[21] Isles MG, McConkey C, Mehanna HM (2008). A systematic review and meta‐analysis of the role of positron emission tomography in the follow-up of head and neck squamous cell carcinoma following radiotherapy or chemoradiotherapy. Clin Otolaryngol.

[22] Porceddu SV, Jarmolowski E, Hicks RJ (2005). Utility of positron emission tomography for the detection of disease in residual neck nodes after (chemo) radiotherapy in head and neck cancer. Head Neck.

[23] Greven KM, Williams DW, McGuirt WF (2001). Serial positron emission tomography scans following radiation therapy of patients with head and neck cancer. Head Neck.

[24] Lowe VJ, Boyd JH, Dunphy FR (2000). Surveillance for recurrent head and neck cancer using positron emission tomography. J Clin Oncol.

[25] Kubota K, Yokoyama J, Yamaguchi K (2004). FDG-PET delayed imaging for the detection of head and neck cancer recurrence after radio-chemotherapy: comparison with MRI/CT. Eur J Nucl Med Mol Imaging.

[26] Lee N, Chuang C, Quivey JM (2002). Skin toxicity due to intensity-modulated radiotherapy for head-and-neck carcinoma. Int J Radiat Oncol Biol Phys.

[27] Vera‐Llonch M, Oster G, Hagiwara M (2006). Oral mucositis in patients undergoing radiation treatment for head and neck carcinoma. Cancer.

[28] Shankar LK, Hoffman JM, Bacharach S (2006). Consensus recommendations for the use of 18F-FDG PET as an indicator of therapeutic response in patients in National Cancer Institute Trials. J Nucl Med.

[29] Humphris GM, Rogers S, McNally D (2003). Fear of recurrence and possible cases of anxiety and depression in orofacial cancer patients. Int J Oral Maxillofac Surg.

[30] Katz MR, Irish JC, Devins GM (2003). Psychosocial adjustment in head and neck cancer: the impact of disfigurement, gender and social support. Head Neck.

